# Psychometric properties of the Flourishing scale in Greek adult population

**DOI:** 10.1186/s40359-026-04378-9

**Published:** 2026-03-19

**Authors:** Panagiota Alifragki, Valia Baralou, Giota Touloumi

**Affiliations:** Department of Hygiene, Epidemiology and Medical Statistics, Medical School, National and Kapodistrian University of Athens, Athens, Greece

**Keywords:** Well-being, Flourishing Scale, reliability, validity, Greek

## Abstract

**Background:**

Well-being is a crucial indicator of individuals’ overall health, encompassing both psychological functioning and life satisfaction. The Flourishing Scale (FS), a brief yet comprehensive measure of well-being, is widely employed in both research and clinical settings to assess psychological flourishing. However, its test-retest reliability has not been evaluated in Greece. This study aims to examine the test-retest reliability and validity of the FS within the Greek context.

**Methods:**

The psychometric properties of the FS were assessed in a sample of 200 Greek adult participants and in a randomly chosen and representative sample of adult population in Greece (data from the National Health Examination Survey: EMENO). Test-retest reliability was evaluated using Intraclass Correlation Coefficient (ICC), while Cronbach’s α and McDonald’s omega were employed to assess internal consistency. Both Exploratory Factor Analysis (EFA) and Confirmatory Factor Analysis (CFA) were conducted to explore and confirm the factor structure of the FS. Test-retest reliability, internal consistency (Cronbach’s alpha) and measurement invariance were further investigated across different demographic groups.

**Results:**

FS demonstrated robust reliability in the Greek sample. The overall Cronbach’s α was 0.84, indicating good internal consistency, while McDonald’s omega total was 0.85, supporting the scale’s reliability. The ICC values for the FS total score and individual items were all above 0.6, suggesting a satisfactory level of repeatability, with several items achieving excellent levels of test-retest reliability. The results from both the EFA and CFA supported a one-factor model for the FS.

**Conclusions:**

The Flourishing Scale (FS) is a reliable and valid instrument for assessing well-being in the Greek context. The scale exhibits strong psychometric properties, including high internal consistency and test-retest reliability, making it a valuable tool for both research and practical applications within this cultural context.

**Supplementary Information:**

The online version contains supplementary material available at 10.1186/s40359-026-04378-9.

## Introduction

Well-being is a multifaceted construct that serves as a key indicator of an individual’s overall health and quality of life. It reflects psychological well-being and life satisfaction, making it a crucial measure in both public health and psychology [1, 2]. According to the World Health Organization [3], health is not merely defined as the absence of disease or infirmity but as a state of complete physical, mental, and social well-being. This broader understanding of health emphasizes the significance of well-being in holistic health assessments, where subjective experiences and emotional states are just as important as objective measures of physical health.

The concept of well-being can be broken down into several dimensions. First, it includes an evaluative component, where individuals assess their life satisfaction and happiness. Second, it encompasses affective aspects, such as emotions like sadness, anger, and anxiety that arise in daily life. Lastly, well-being also includes cognitive judgments regarding the meaning and purpose of life [4]. These components highlight the complexity of well-being and the necessity of accurate, reliable measurement tools.

A key distinction in the study of well-being is between objective well-being, which refers to externally measurable factors such as income or health status, and subjective well-being, which is based on personal judgments and emotional reactions. Subjective well-being is often characterized by self-reported satisfaction with life and the balance between positive and negative emotions [4]. Given the importance of well-being for individuals and societies, there is a growing need for reliable and valid instruments to assess it accurately, as such assessments can inform both policy and individual-level interventions [5].

Although research on well-being has traditionally focused on hedonic aspects such as life satisfaction and positive affect [6, 7], recent work emphasizes the significance of flourishing, which encompasses optimal functioning, personal growth, and the realization of human potential [8,9]. Flourishing is conceptually related to, yet distinct from, well-being, as it highlights not only subjective happiness but also social and psychological functioning. Explicitly addressing flourishing provides a more comprehensive framework for understanding positive mental health and factors contributing to optimal human development.

Various scales have been developed to measure well-being and its components. For instance, the Scale of Positive and Negative Experience (SPANE) captures both positive and negative subjective feelings [8, 9], while the Satisfaction with Life Scale (SWLS) assesses overall life satisfaction [10]. Another notable instrument is the Sovereign Wellbeing Index, which incorporates many elements from these scales and offers a comprehensive assessment of well-being [11] .

A scale that has gained considerable attention is the Flourishing Scale (FS), a brief yet powerful instrument designed to assess how individuals perceive their success in essential life domains, such as relationships, self-esteem, purpose, and optimism [8]. Its widespread use in research can be attributed to its strong psychometric properties, as well as its simplicity and efficiency in assessing key dimensions of psychological well-being. The FS has demonstrated good reliability and validity across various populations and has been translated into multiple languages, including Italian [12], Indian [13], Portuguese [14], Japanese [15], and Iranian [16]. These studies consistently support its one-factor structure, which aligns with the conceptualization of flourishing as a unified construct of psychological well-being.

Both validity and reliability are fundamental psychometric properties that determine the quality and utility of a questionnaire. Validity pertains the extent to which an instrument accurately measures the construct it is intended to assess [17] while reliability refers to the consistency of a measure and is essential for ensuring that an instrument produces stable and accurate results over time. Two primary forms of reliability are internal consistency, which reflects how well the items of a scale correlate with each other, and test-retest reliability, which measures the stability of the instrument across different time points [18, 19]. In the context of psychometric scales, high test-retest reliability suggests that the measure can reliably capture a stable construct over time, making it a critical property for any psychological scale [20].

Diener et al. [8] evaluated the test-retest reliability of the FS in their original study, reporting strong results, which suggested that the scale has good temporal stability. Similarly, Sumi [15] assessed the test-retest reliability of the FS in Japan and confirmed its consistency across time. These findings highlight the importance of this psychometric property, especially for instruments that measure constructs like well-being, which are expected to remain relatively stable in the absence of significant life changes or interventions.

In the Greek context, previous studies have examined the Flourishing Scale (FS) among adults, including a large-scale validation study based on a sample of 2,272 participants, which confirmed its factorial structure and internal consistency [21]. Nevertheless, gaps remain regarding the stability of the scale over time and the potential influence of demographic factors on its measurement properties. Addressing these gaps is essential to ensure that the FS provides reliable and generalizable results across different subgroups. Moreover, although previous studies employed relatively large samples, they were not based on nationally representative population data, which may limit the generalizability of the findings at the population level.

Demographic characteristics such as gender, age, and place of residence have been shown to significantly impact mental health and flourishing [22–24]. These variables may also affect the stability of responses over time, highlighting the importance of examining test–retest reliability of the FS across these groups. Finally, while Cronbach’s alpha is typically reported for the total sample, examining alpha coefficients separately by groups (e.g., gender, age group, residence or time of measurement) has not been explored in the Greek population and allows researchers to evaluate whether the scale demonstrates comparable reliability across populations.

Building on this background, the present study extends previous research by providing a comprehensive psychometric evaluation of the Flourishing Scale using data from the nationally representative EMENO health examination survey, assessing its internal consistency across key demographic groups (gender, age, and place of residence) and evaluating its measurement invariance over time. By integrating these aspects and leveraging a large, nationally representative sample, this study seeks to provide comprehensive evidence on the temporal stability and cross-group reliability of the FS, thereby enhancing its applicability and psychometric robustness within the Greek cultural context.

## Methods

### Participants

The present research was structured into two complementary studies. Study 1, based on a large sample, examined the factorial structure and measurement invariance of the Flourishing Scale, whereas Study 2, that included a smaller sample, focused on reliability and supplementary validity assessment.

In study 1, the data for the testing of CFA and EFA were obtained from the larger sample of the EMENO health examination survey, a cross-sectional, population-based study conducted in Greece involving 6006 participants. The study focused on a randomly selected sample based on the 2011 census that represented adult population aged ≥ 18 years and covered urban, semi-urban, and rural areas. In practice, regions with larger populations were underrepresented, while regions with smaller populations were overrepresented. To account for this in the statistical analysis of the general population data, the sampling design of the study was taken into consideration, using appropriate weighting. Typically, in cross-sectional health surveys, despite good design, women and older age groups are often overrepresented in the final sample. To address this, additional weighting was applied so that the sample’s age, gender, and geographic distribution closely resembled that of the general adult population living in Greece, based on the 2011 census from ELSTAT. The full details of the EMENO health examination study can be found in previously published report and protocol [25]. Ethics approval and consent to participate in the EMENO study was approved by the Ethics and Deontology Committee of the National and Kapodistrian University of Athens (Date: 8 November 2012, Protocol number: 1742) and by the Hellenic Data Protection Authority (Date: 7 December 2012, Protocol number: GN/ΕX/1069-1/07-12-2012). A modified version of the informed consent form (ICF) was approved by the Ethics and Deontology Committee of the National and Kapodistrian University of Athens (Date: 6 March 2013, Protocol number: 6315).

In study 2, to assess the test-retest reliability of the Flourishing Scale (FS), a sample of 200 adults residing in Greece was collected using a non-probabilistic snowball sampling method. This technique, advantageous for tapping into social networks and ensuring access to a range of demographics [26], facilitated the inclusion of individuals from various ages, areas, educational, and socioeconomic backgrounds. All participants provided informed consent and were briefed on the purpose and voluntary nature of the study, in accordance with ethical standards as outlined by the Declaration of Helsinki [27]. Study 2 has been approved by the ethics and deontology committee of the Medical School of the National and Kapodistrian University of Athens (078/25.02.2019). Eligibility criteria were minimal to ensure a broad, inclusive sample, encompassing only the requirement that participants were at least 18 years of age [28].

### Sample size

In Study 1, data from 6,006 adults drawn from the nationally representative EMENO health examination survey were used to conduct exploratory and confirmatory factor analyses (EFA and CFA) and to test measurement invariance across demographic groups. This sample size substantially exceeds commonly recommended thresholds for factor analytic studies and ensures high statistical power, stable parameter estimates, and robust evaluation of the scale’s factorial validity.

Methodological guidelines indicate that sample sizes ranging from 100 to 200 participants are sufficient for estimating reliability indices such as Cronbach’s alpha, McDonald’s omega, and intraclass correlation coefficients (ICC), particularly for unidimensional scales with a limited number of items [29–31]. Furthermore, for test–retest reliability, samples of at least 50–100 participants are generally considered adequate, with larger samples providing greater precision and narrower confidence intervals for ICC estimates [32]. Therefore, the sample size of 200 adults employed in Study 2 was deemed methodologically appropriate.

### Measures

The Flourishing Scale [8] was used to assess participants’ psychological flourishing. The scale has been previously developed and validated in English. The FS was adapted for use in the Greek context by Kyriazos et al. [21] and it was administered using the Greek version [33]. The translation followed the standard forward translation and back-translation procedure described by Brislin [34], involving independent bilingual translators and resolution of discrepancies to ensure semantic and conceptual equivalence with the original instrument. The scale consists of eight items, each rated on a 7-point Likert scale ranging from 1 (strongly disagree) to 7 (strongly agree), resulting in scores between 8 and 56. High scores indicate elevated levels of self-perceived flourishing, encompassing psychological strengths such as purpose, competence, and positive relationships [8]. The eight items are: “I lead a purposeful and meaningful life,” “My social relationships are supportive and rewarding,” “I am engaged and interested in my daily activities,” “I actively contribute to the happiness and well-being of others,” “I am competent and capable in the activities that are important to me,” “I am a good person and live a good life,” “I am optimistic about my future,” and “People respect me” [8]. In this study, participants also provided demographic information (gender, age, educational level, study institution, year of study) and reported on any chronic health conditions and subjective health status to investigate potential influences on their responses.

### Procedure

For the data used for the EFA (Study 1), the sample was collected between May 2013 and June 2016. This survey was carried out through “door-to-door” interviews, using Computer Assisted Personal Interviews (CAPI), along with physical examinations and additional data collection by trained professionals, including physicians and researchers. Concerning the test-retest analysis (Study 2) the data collection spanned January to April 2019, during which participants completed the FS-G twice, with a two-week interval to assess temporal stability. Phase I included the administration of the FS-G and a demographic questionnaire. Two weeks later at Phase II, participants were additionally asked whether any significant emotional or life events had occurred since the first assessment, aiming to identify external influences on changes in their scores [35].

### Statistical analysis

For Study 1, weighted summary statistics were provided; sampling weights to adjust for study design were multiplied by post-stratification weights to match the sample age, sex and geographical distribution to that of the general population based on the 2011 census. Consistent with best practice recommendations for scale validation [36, 37], a split-sample cross-validation approach was applied in Study 1. The total sample was randomly divided into two subsamples of equal size. An Exploratory Factor Analysis (EFA) was conducted in the first subsample to examine the underlying factor structure of the Flourishing Scale. EFA with principal axis factoring was applied to explore the latent structure of the FS-G without pre-imposed constraints [38]. To perform EFA, the factorability of the data was first tested using the Kaiser-Meyer-Olkin (KMO) measure and Bartlett’s Test of Sphericity. A KMO value ≥ 0.60 indicates appropriate data for factor analysis, a value closer to 1.0 indicates that the variables are highly factorable, while values closer to 0.0 suggest that the data may not be appropriate for factor extraction [39]. Bartlett’s test was conducted to assess the adequacy of correlations between variables, with a significance threshold set at *p* < 0.05 [40]. The factor loadings were also evaluated. Specifically, values ≥ 0.40 are considered meaningful, and values above 0.70 are ideal [41] while factor loadings below 0.40 suggest weak associations. In addition, a scree plot was used to determine the number of factors to retain by plotting eigenvalues against factor numbers. The point where the eigenvalues begin to level off (the elbow) indicates the number of factors to keep. Factors before the elbow represent significant variance, while those after the elbow explain minimal additional variance and can be discarded. A common rule is to retain factors with eigenvalues greater than 1 [39]. The scree plot is a valuable tool for ensuring that the model retains only meaningful factors while avoiding overfitting [42].

Subsequently, a Confirmatory Factor Analysis (CFA) was performed in the second subsample of Study 1 to test whether the factor structure identified in the EFA could be replicated. Factor loadings represent the strength of the relationship between observed variables and their underlying latent construct and ideally should be ≥ 0.70, indicating a strong and reliable association [43]. Loadings between 0.40 and 0.70 are acceptable. The same applies to standardized factor loadings. Furthermore, all factor loadings should be statistically significant (*p* < 0.05), which supports their inclusion as valid indicators of the factor. In addition to examining factor loadings, several goodness-of-fit indices were employed to assess the overall fit of the CFA model. Among fit indices, the Chi-Square (χ²) test evaluates the discrepancy between the observed and model-implied covariance matrices, with a non-significant result (*p* > 0.05) indicating a good fit [44]. However, due to its sensitivity to sample size, we also used the following additional indices: the Standardized Root Mean Square Residual (SRMR), the Root Mean Square Error of Approximation (RMSEA), the Comparative Fit Index (CFI) and the Tucker-Lewis Index (TLI). SRMR values ≤ 0.08 and RMSEA values ≤ 0.06 indicate excellent fit, while values up to 0.08 may still be considered acceptable [45] and values between 0.08 and 0.10 typically indicate an acceptable, though not optimal fit [46]. As for the CFI and TLI, values ≥ 0.95 reflect excellent fit and values ≥ 0.90 are considered acceptable [46]. Measurement invariance of the Flourishing Scale was examined in Study 1 across gender, place of residence, and age groups using multi-group confirmatory factor analysis (MGCFA). A hierarchical sequence of increasingly constrained models was tested: configural invariance (same factor structure across groups), metric invariance (equal factor loadings), scalar invariance (equal factor loadings and thresholds), and strict invariance (equal factor loadings, thresholds, and residual variances). Model fit was evaluated using the CFI, TLI, RMSEA, and SRMR indices. Invariance was assessed based on changes in fit indices between nested models, with ΔCFI (change in Comparative Fit Index) ≤ 0.010 and ΔRMSEA (change in Root Mean Square Error of Approximation) ≤ 0.015 indicating acceptable invariance [47].

For Study 2, descriptive statistics were used to summarize the sample characteristics (Table S2) and item distributions since the item scores were not normally distributed (Table S3, Table S4). Internal consistency was assessed with Cronbach’s alpha [48] and McDonald’s omega [49]. The benchmarks for Cronbach’s alpha were set following Nunnally’s [48] recommendation and they suggest that an alpha value of 0.70 or higher is considered acceptable for exploratory research, a threshold of 0.80 or higher indicates good reliability for established measures and finally in cases requiring critical evaluations or precise assessments, a stricter benchmark of 0.90 or higher is often required. McDonald’s ω was interpreted using commonly accepted psychometric conventions, whereby values of 0.70 indicate acceptable reliability, values of 0.80 indicate good reliability, and values of 0.90 or higher indicate excellent internal consistency [49–51]. Test-retest reliability was evaluated using the Intraclass Correlation Coefficient (ICC). The ICC serves as an index of stability for repeated measures, ranging from 0 to 1 [20], with higher values indicating superior reliability. Classifications of the ICC are typically categorized as poor (ICC < 0.5), moderate (0.5 ≤ ICC < 0.75), good (0.75 ≤ ICC < 0.9), and excellent (ICC ≥ 0.9) [32].

The statistical analysis was performed using SPSS version 17 and R version 4.02, following rigorous statistical practices [52, 53].

## Results

### Sample characteristics

Study 1 consisted of 6006 individuals participated in the Greek Health Examination Survey, EMENO, with an overall response rate of 72%. Of these, 13 had missing values for age and gender. In addition, participants who were missing ≥ 6 well-being questions from the FS scale out of the 8 questions (218 participants) were excluded from the analysis, yielding a total of 5782 study participants. For those missing up to 5 questions, the missing values were imputed using the mean values from the other responses. Basic demographic characteristics of Study 1 are shown in Table S1. In Study 1, the weighted mean well-being score measured using the FS scale was 47.14, with a standard deviation of 7.18. As expected, the maximum and minimum scores were 56 and 8, respectively, with the median score being 48.

Study 2 had no missing responses. Demographic characteristics of Study 2 sample are presented in detail in Table 1. Study 2 included 108 females (54%), aged from 20 to 91 years. The distribution by residence showed that most respondents (71%) were from urban areas. In terms of health status, 74.5% of participants reported no chronic disorders. Additionally, 87% of the sample assessed their subjective health status as good or very good, indicating a generally healthy cohort. The median FS total score for Phase I was 49 (Q1-Q3 = 6.5), with individuals’ scores ranging from 33 to 56 (Table S2). For Phase II, the median FS total score was 49 (Q1-Q3 = 7), with a range from 32 to 56 (Table S2). A Shapiro-Wilk normality test was conducted on the data for both phases, and the results indicated that in both cases, the data were not normally distributed. This finding is also graphically confirmed in the Figures S1& S2 of the Supplementary Material. Additional descriptive statistics of the total FS score for both Phases are provided in Table S2 (Supplementary Material). The participants tended to respond mostly positively, resulting in a distribution curve that is shifted to the right. The frequencies of responses for each FS item across both phases are presented in detail in Tables S2 & S3 of the Supplementary Material; providing a granular view of response patterns.

**Table 1:** Demographic characteristics of Study 2 (Total *n* = 200)

	n (%)
Gender	
Female	108 (54)
Male	92 (46)
Residential area	
Urban	142 (71)
Non-urban	58 (29)
Chronic Disorder	
Yes	43 (21.5)
No	149 (74.5)
I don’t know	6 (3)
Prefer not to answer	2 (1)
Subjective health estimation	
Very good	82 (41)
Good	92 (46)
Moderate	22 (11)
Bad	1 (0.5)
Very bad	2 (1)
Don’t know	1 (0.5)
Prefer not to answer	0 (0)
Age group (years)	
18–24	39 (19.5)
25–29	29 (14.5)
30+	132 (66)

### Exploratory factor analysis

An Exploratory Factor Analysis (EFA) was conducted using Principal Axis Factoring (PAF) with oblimin rotation on one randomly selected half sample of Study 1 following a split-sample procedure. Before the analysis, the data were assessed for factorability, yielding a ΚΜΟ measure of sampling adequacy of 0.91, which is above the acceptable threshold, ensuring reliable factor extraction [39]. Additionally, Bartlett’s Test of Sphericity was significant (χ² = 24269.35, *p* < 0.001), confirming that the correlation matrix was not an identity matrix and hence, the variables were suitable for factor analysis. The EFA revealed a dominant single factor with an eigenvalue of 4.18, accounting for 52% of the total variance in the Flourishing Scale items. The scree plot (Figure S3 in the supplementary material) showed a distinct decline after the first component, reinforcing the decision to retain only one factor. Factor loadings were statistically significant and ranged from 0.58 to 0.83 (Table 2), demonstrating most of the items contributed meaningfully to the underlying construct of psychological well-being. Parallel Analysis was also conducted to determine the optimal number of factors (Figure S3 in the supplementary material). The first factor had an eigenvalue well above the simulated random data, confirming it as a robust factor. The second factor fell below the simulated eigenvalue curve and was therefore not considered strong. All subsequent factors had eigenvalues below 1 and below the random data thresholds. Thus, Parallel Analysis supports a unidimensional structure, suggesting the retention of a single factor, consistent with the findings from the Scree Plot. These results reinforce the unidimensional nature of the Flourishing Scale across both phases and highlight its consistency as a reliable measure of psychological well-being.

**Table 2:** Factor loadings from EFA of the *FS* items, Study 1

Flourishing Scale item	Factor loadings	
Q6. My social relationships are supportive and rewarding		0.72
Q5. I am competent and capable in the activities that are important to me		0.80
Q4. I am engaged and interested in my daily activities		0.79
Q1. I am a good person and lead a good life		0.73
Q3. I lead a purposeful and meaningful life		0.83
Q2. I am optimistic about my future		0.64
Q7. I actively contribute to the happiness and wellbeing of others		0.66
Q8. People respect me		0.58

FS Flourishing Scale. Extraction method: Principal Axis Factoring (PAF)

### Confirmatory factor analysis

**Fig. 1:**
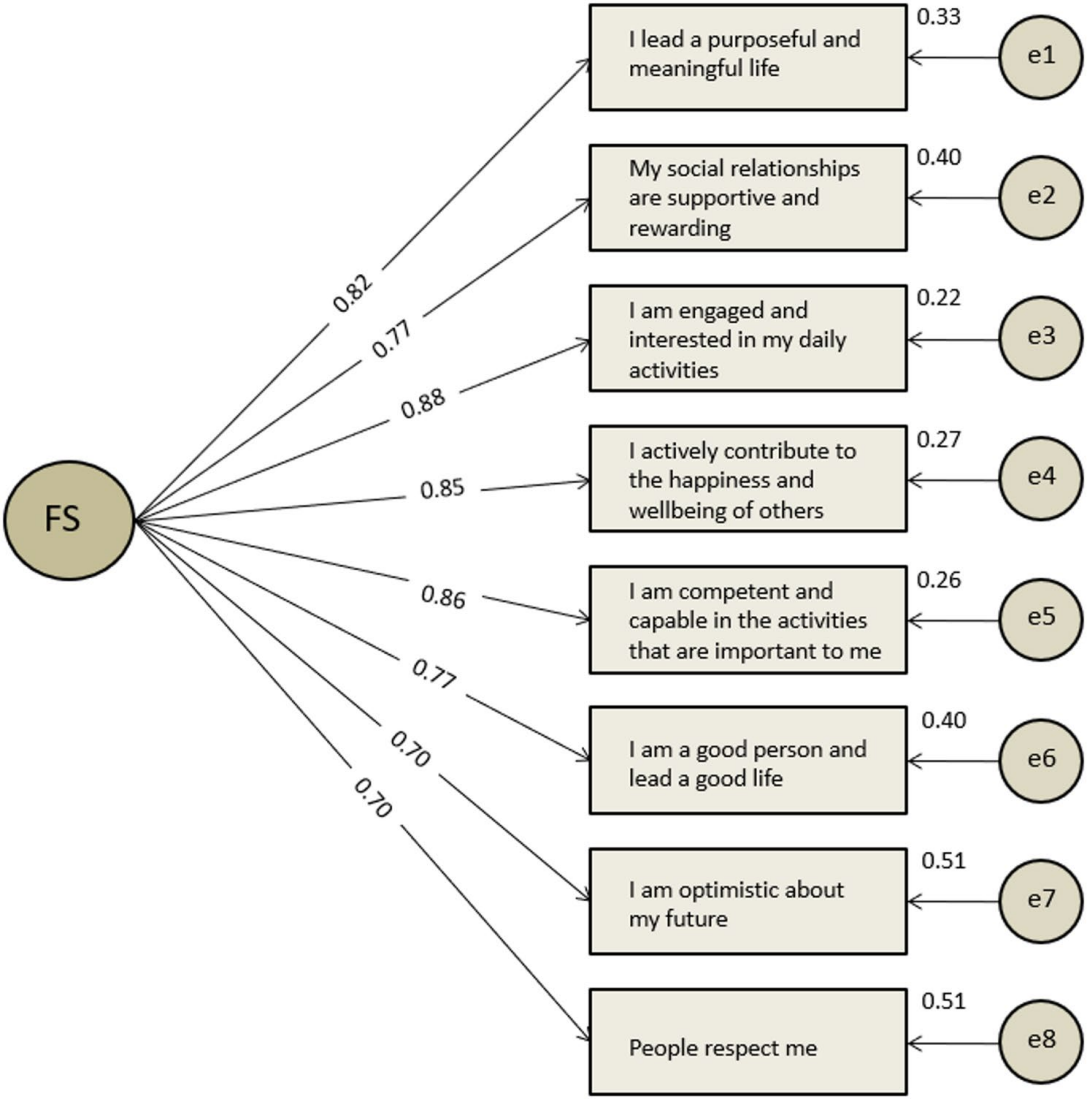
Confirmatory Factor Analysis results for one-factor model of Study 1. FS = Latent factor representing well-being. Numbers on arrows from FS to items represent standardized factor loadings. ei, i[1,8] = Numbers on arrows from circles to items are errors that show unexplained variance for each item

To test the unidimensional structure of the FS, a Confirmatory Factor Analysis (CFA) was conducted using the remaining portion of the split sample of Study 1. Given that the items were ordinal (1–7 Likert scale), the analysis employed the Weighted Least Squares Mean and Variance adjusted (WLSMV) estimator, which is appropriate for ordinal data [42]. The results demonstrated strong model fit with indices of CFI = 0.99, TLI = 0.99, and RMSEA = 0.09, SRMR = 0.041. Although RMSEA was slightly above the conventional threshold, other fit indices indicated an excellent model fit which accurately represents the data, adhering to the criteria for a well-fitting unidimensional structure. In addition, all factor loadings were high, exceeding 0.7 and statistically significant (*p* < 0.001), supporting their inclusion as valid indicators of the flourishing factor (Fig. 1).

### Measurement invariance across demographic groups

Measurement invariance of the Flourishing Scale was examined across gender, residential area, and age groups in Study 1 using multi-group confirmatory factor analysis with ordinal indicators and WLSMV estimation. A hierarchical sequence of configural, metric, scalar, and strict models was tested (Table 3). Across all grouping variables, the configural models demonstrated CFI and TLI values equal to or greater than 0.993 and SRMR values ranging from 0.041 to 0.043, all meeting recommended criteria. RMSEA values for configural models ranged from 0.092 to 0.095, falling within the range typically considered acceptable. Transitions from configural to metric, scalar, and strict models were associated with negligible changes in model fit. Across all comparisons, changes in CFI ranged from − 0.002 to 0.001, and changes in RMSEA ranged from − 0.029 to 0.001. All changes were well below the recommended thresholds (ΔCFI ≤ 0.010; ΔRMSEA ≤ 0.015). Importantly, comparisons between scalar and strict models showed negligible changes in fit (ΔCFI ≤ 0.001; ΔRMSEA ≤ 0.003 across grouping variables). Thus, strict measurement invariance was achieved across all examined demographic groups.

**Table 3:** Measurement invariance fit indices for the Flourishing Scale across gender, residential area and age groups in Study 1

Grouping variable	Model	CFI	TLI	RMSEA	SRMR	ΔCFI	ΔRMSEA
Gender	Configural	0.995	0.993	0.092	0.041	-	-
	Metric	0.995	0.994	0.087	0.042	0.000	-0.005
	Scalar	0.995	0.997	0.065	0.041	0.000	-0.022
	Strict	0.995	0.997	0.065	0.041	0.000	0.000
Residential area	Configural	0.995	0.993	0.095	0.043	-	-
	Metric	0.995	0.994	0.089	0.045	0.000	-0.006
	Scalar	0.995	0.997	0.062	0.043	0.000	-0.027
	Strict	0.995	0.997	0.062	0.043	0.000	0.000
Age groups	Configural	0.995	0.993	0.093	0.043	-	-
	Metric	0.993	0.993	0.094	0.048	-0.002	0.001
	Scalar	0.994	0.996	0.065	0.043	0.001	-0.029
	Strict	0.995	0.997	0.062	0.043	0.001	-0.003

*CFI* = Comparative Fit Index; *TLI* = Tucker-Lewis Index; *RMSEA* = Root Mean Square Error of Approximation; *SRMR* = Standardized Root Mean Square Residual; ΔCFI and ΔRMSEA represent changes between successive models

### Reliability

Reliability was assessed in Study 2, which was conducted in two Phases, as reported. For Phase I the overall Cronbach’s alpha (α) was 0.80, 95% CI (0.76, 0.84). The Cronbach’s alphas if item dropped varied from 0.76 to 0.81, which are acceptable values for internal consistency [17]. Corrected item-total correlations (i.e., correlations between the respective item and the total FS sum score without the respective item) ranged from 0.35 to 0.70. Consistent with these findings, McDonald’s omega total for the scale was 0.81, further supporting good internal consistency. Omega total values remained stable when individual items were removed from 0.78 to 0.82, indicating that no single item disproportionately influenced the overall reliability [49]. As for reliability in Phase II, the overall Cronbach’s alpha (α) was 0.84, 95% CI (0.81, 0.87). The Cronbach’s alphas if item dropped varied from 0.81 to 0.85, which are acceptable values for internal consistency. Corrected item-total correlations ranged from 0.41 to 0.73. Consistent with these findings, McDonald’s omega total was 0.85, further supporting good internal consistency of the scale. Omega total values remained stable when individual items were removed (range: 0.82–0.85), suggesting that no single item disproportionately influenced the overall reliability. Internal consistency of the Flourishing Scale (FS) was examined across key demographic groups and between the two measurement occasions. Regarding age, Cronbach’s alpha values were 0.867 for participants aged 18–24 years, 0.840 for those aged 25–29 years, and 0.763 for participants aged 30 years and above. Feldt’s tests indicated no statistically significant differences between the 18–24 and 25–29 groups or between the 18–24 and 30 + groups (*p* > 0.05), whereas a significant difference emerged between the 25–29 and 30 + groups (F = 1.776, *p* = 0.034), suggesting slightly lower internal consistency among the older participants. For gender, alpha values were 0.807 for males and 0.802 for females, with no significant difference observed (F = 1.03, *p* = 0.899). Regarding area of residence, alpha was 0.811 for urban and 0.776 for non-urban participants, with no significant difference (F = 1.18, *p* = 0.475). Finally, Cronbach’s alpha increased from 0.80 to 0.84 between the two measurement occasions, and the difference was statistically significant (*t*(198) = 2.57, *p* = 0.011), indicating that the scale’s internal consistency slightly improved over time.

As for test-retest reliability the overall ICC was 0.89 (95% CI: 0.85,0.91), with subgroup ICC values ranging from 0.82 to 0.96, indicating good correlation values. Item-level ICC values ranged from 0.63 to 0.83 for the full sample, suggesting at least moderate degree of test-retest reliability. The overall and item specific ICC values for the whole sample as well as across specific subgroups categorized by age, gender, and place of residence are presented in detail in Table 4. It is worth noting though that certain items, such as item 1 (“I lead a purposeful and meaningful life”), item 3 (“I am engaged and interested in my daily activities”), item 5 (“I am optimistic about my future”) and item 7(“I actively contribute to the happiness and wellbeing of others”) achieved quite good stability for the whole sample with ICCs greater than 0.75. The reliability assessment of the specific items across different subgroups demonstrated that most cases exhibited moderate to good stability. Item 1 and 5 have good reliability across all subgroups. However, only ICC of item 2 (“My social relationships are supportive and rewarding”) is poor (< 0.5), particularly among male participants, non-urban residents, and individuals over 30 years old. Items 3 (“I am engaged and interested in my daily activities”) and 8 (“People respect me”) similarly displayed reduced test-retest reliability when analyzed within the non-urban subgroup.

**Table 4:** Total and item specific intraclass coefficients ICC and 95% confidence intervals for Study 2 and by subgroup characteristic

Item	Full sample (*N* = 200)	Males (*N* = 92)	Females (*N* = 108)	Urban (*N* = 142)	Non-urban (*N* = 58)	18–24 (*N* = 39)	25–29 (*N* = 29)	30+ (*N* = 132)
1	0.81 (0.75, 0.86)	0.81 (0.71, 0.87)	0.82 (0.74, 0.88)	0.75 (0.66, 0.82)	0.89 (0.82, 0.94)	0.77 (0.57, 0.88)	0.80 (0.58, 0.91)	0.82 (0.74, 0.87)
2	0.63 (0.52, 0.72)	0.53 (0.29, 0.69)	0.75 (0.63, 0.83)	0.68 (0.56, 0.77)	0.45 (0.07, 0.68)	0.77 (0.56, 0.88)	0.84 (0.65, 0.92)	0.57 (0.40, 0.70)
3	0.80 (0.74, 0.85)	0.83 (0.75, 0.89)	0.78 (0.68, 0.85)	0.84 (0.78, 0.89)	0.52 (0.19, 0.72)	0.87 (0.75, 0.93)	0.85 (0.66, 0.93)	0.74 (0.64, 0.82)
4	0.74 (0.66, 0.80)	0.62 (0.43, 0.75)	0.83 (0.75, 0.88)	0.74 (0.63, 0.81)	0.72 (0.53, 0.84)	0.86 (0.74, 0.93)	0.67 (0.28, 0.84)	0.68 (0.55, 0.78)
5	0.83 (0.77, 0.87)	0.88 (0.82, 0.92)	0.77 (0.66, 0.84)	0.83 (0.76, 0.88)	0.82 (0.70, 0.89)	0.80 (0.62, 0.90)	0.92 (0.83, 0.96)	0.80 (0.72, 0.86)
6	0.76 (0.69, 0.82)	0.80 (0.70, 0.87)	0.72 (0.59, 0.81)	0.76 (0.66, 0.83)	0.76 (0.59, 0.86)	0.75 (0.52, 0.87)	0.82 (0.62, 0.92)	0.74 (0.63, 0.81)
7	0.75 (0.75, 0.81)	0.74 (0.61, 0.83)	0.77 (0.66, 0.84)	0.79 (0.71, 0.85)	0.65 (0.41, 0.80)	0.93 (0.86, 0.96)	0.93 (0.86, 0.97)	0.66 (0.52, 0.76)
8	0.72 (0.63, 0.79)	0.61 (0.41, 0.74)	0.83 (0.74, 0.88)	0.78 (0.69, 0.84)	0.58 (0.29, 0.76)	0.71 (0.45, 0.84)	0.87 (0.73, 0.94)	0.69 (0.56, 0.78)
Overall	0.89 (0.85,0.91)	0.87 (0.80, 0.91)	0.90 (0.85, 0.93)	0.90 (0.93, 0.96)	0.82 (0.70, 0.90)	0.93 (0.87, 0.96)	0.96 (0.92, 0.98)	0.83 (0.76, 0.88)

## Discussion

The present study provided evidence for the reliability and factorial validity of the FS within the Greek context. The findings from both the EFA and CFA analyses supported a one-factor model for the FS. These findings underscore the FS’s robustness as a consistent measure of well-being across varied populations and time periods. In addition, participants generally perceived themselves positively in the key domains of psychological and social functioning, which is consistent with findings from other cultural adaptations [54]. The results indicate that the FS demonstrates robust reliability in the Greek sample. The overall Cronbach’s α indicating good internal consistency. The ICC values for the FS total score and the individual items were all suggesting a satisfactory level of repeatability, with several items achieving excellent levels of test-retest reliability.

Exploratory and confirmatory factor analyses conducted on the split data set further reinforced the unidimensional nature of the FS, aligning with previous studies conducted in different cultural contexts [8, 14, 15, 21]. The results of this study revealed stability in mean FS scores and consistent self-perceptions of well-being among participants across the two assessment points. Overall, our findings corroborate the FS’s capacity to measure a singular, cohesive construct of psychological well-being. The use of a split-sample cross-validation design constitutes a methodological strength of the present study. By conducting an Exploratory Factor Analysis (EFA) in one subsample and a Confirmatory Factor Analysis (CFA) in an independent subsample, the risk of overfitting was minimized and the stability of the factor structure was more rigorously assessed. Such an approach has been widely recommended in the scale development and validation literature as a means of enhancing the robustness and replicability of findings [38, 55]. This strengthens the evidence for the unidimensional structure of the Flourishing Scale and supports its applicability across diverse populations. Although CFI and TLI indicated excellent model fit, the RMSEA value was relatively elevated and should be interpreted with caution. RMSEA is known to perform less reliably in models with few degrees of freedom and in large samples, particularly in simple unidimensional models with a small number of observed variables [56]. Given that the Flourishing Scale comprises a single factor with eight items, the inflated RMSEA estimate is likely attributable to model characteristics rather than poor fit. In contrast, the SRMR value (0.041) indicated good model fit and may be a more appropriate index in this context.

Measurement invariance analyses across gender, place of residence and age supported configural, metric, and scalar invariance, indicating that the Flourishing Scale assesses the construct of flourishing equivalently across these demographic groups. Furthermore, the establishment of strict measurement invariance suggests that observed differences in flourishing scores can be meaningfully interpreted as true group differences rather than measurement artifacts [57]. Converging evidence from fit indices indicated good to excellent model fit across all invariance models. The relatively elevated RMSEA values observed in the configural and metric models should be interpreted with caution, given the documented sensitivity of RMSEA to low degrees of freedom in multigroup CFA [47, 56]. The demonstration of this highest level of invariance provides strong evidence for the robustness of the Flourishing Scale and supports its use in both research and applied contexts involving heterogeneous populations [58, 59]. These findings reinforce the comparability of scores and the appropriateness of the FS for use in diverse subpopulations, highlighting its reliability and validity in the Greek context.

The overall Cronbach’s alpha (α) and McDonald’s omega for the FS suggested high internal consistency observed surpasses the thresholds recommended [60, 61], indicating that the FS-G is a reliable tool for measuring well-being within this population. In addition, examining Cronbach’s alpha across groups provided a more nuanced understanding of the scale’s reliability. The results indicate that the FS demonstrates high internal consistency across different demographic groups, with only minor variations among age groups. The slightly lower alpha in participants aged 30 + may reflect greater heterogeneity in flourishing perceptions in this age range. The absence of significant differences by gender or area of residence suggests that the FS operates consistently across these groups. Importantly, the significant increase in Cronbach’s alpha between the two measurement occasions demonstrates that the scale is not only stable over time but may also benefit from increased participant familiarity or improved understanding of items, leading to more homogeneous responses. Overall, these findings support the reliability and robustness of the FS for use in diverse adult populations and across repeated assessments. Reporting group-specific reliability contributes to transparency and facilitates future meta-analytic efforts in reliability generalization [62]. Furthermore, it supports the psychometric soundness of the instrument across diverse populations, reinforcing confidence in the comparability of results between subgroups. Test-retest reliability for the total sample was satisfactory, with significant stability observed for individual FS items. The test-retest reliability demonstrated through a two-week interval confirms the temporal stability of the FS, aligning with the initial validation study conducted by Diener et al. [8] and subsequent validations in diverse cultural settings such as New Zealand [54], Portugal [14], and Japan [15]. These findings underscore the overall robustness of the FS in capturing consistent assessments of well-being over time, yet they also point to potential variability in specific items based on demographic factors. Such insights could inform future refinements of the scale or contextual adaptations tailored to distinct population groups. However, it is notable that certain subgroups, particularly non-urban residents, demonstrated lower reliability scores. This could be attributed to educational disparities, as indicated by the statistically significant correlation between non-urban residence and educational attainment (χ² = 24.861, *p* < 0.001; data not shown), but it may also indicate that perceptions of social relationships are more context-dependent and variable in these groups, shaped by cultural norms regarding social roles, community structure, and interpersonal expectations. These findings point to potential challenges in understanding certain items within non-urban populations, suggesting the need for tailored strategies to enhance comprehension and ensure accurate responses.

Despite these strengths, this study is also subject to limitations. Firstly, the age range of participants in Study 2 was somewhat narrow, and future studies should assess the FS’s psychometric properties across broader age groups, including Greek adolescents and older adults, to evaluate its generalizability [63]. Secondly, the reliance on self-administered questionnaires, while facilitating ease of data collection, may introduce biases related to participants’ engagement and comprehension levels. Another limitation pertains to the sample composition. The study employed a convenience-based sample, which may limit the external validity of the findings. Although this sampling method enabled efficient recruitment, it poses restrictions on the extent to which these results can be generalized. Future research should strive to include random samples that more accurately represent the general population to enhance the robustness of the findings [63]. The test–retest reliability of the Flourishing Scale was assessed with a sample of 200 participants, which aligns with recommended sample sizes for reliability studies. While this size is sufficient for the overall analysis, reliability estimates within smaller demographic subgroups should be interpreted with caution. Moreover, the factor analyses were based on data from the large, nationally representative EMENO study, which enhances the robustness of the psychometric evaluation and supports the generalizability of the results to the wider Greek adult population.

Τhis study highlights the utility of the FS as a reliable, valid and concise instrument for assessing well-being. Given the adverse economic and social conditions in Greece, particularly during the economic crisis and coronavirus period that profoundly impacted psychological well-being [64], the need for effective tools to measure well-being is increasingly critical. The FS, with its brief format and robust psychometric properties, provides an effective means for tracking well-being and guiding interventions aimed at improving public health.

The implications of using the FS extend beyond individual assessment. Reliable instruments such as the FS are vital for research exploring the relationships between well-being and other psychosocial factors. This can, in turn, inform public health policies and targeted interventions aimed at bolstering psychological resilience and societal support structures. For example, understanding well-being in specific subgroups (e.g., non-urban or less-educated populations) can lead to better-targeted public health strategies and resource allocation. The robustness of the FS demonstrated in this analysis supports its utilization for future research and practical applications within Greek populations and potentially broader demographic groups. The comprehensive fit and meaningful factor loadings contribute to its credibility as a valid and reliable tool for assessing flourishing.

In conclusion, the findings from this study show that the FS is a reliable and valid tool for assessing well-being in Greece, mirroring its utility in other cultural contexts. Continued validation across diverse demographic groups and the inclusion of complementary qualitative methods could further enrich our understanding of well-being dynamics and their broader implications for health and policy.

## Supplementary Information

Supplementary Material 1.

## Data Availability

The datasets used and/or analysed during the current study are available from the corresponding author on reasonable request.
