# Supplementary material for: Psychometric properties of the Flourishing scale in Greek adult population
Source: BMC Psychol. 2026 Mar 19;14:617. doi: 10.1186/s40359-026-04378-9 (PMC13123140; doi:10.1186/s40359-026-04378-9)
Supplement: Supplementary file 1 — Supplementary Material 1. [file 40359_2026_4378_MOESM1_ESM.docx]

**Supplementary material**

**Table S1** Demographic characteristics of the EMENO sample (Study 1) (N=5.782)

|  | | N (%) | | % weighted |
| --- | --- | --- | --- | --- |
| **Gender** |  | |  | |
| Male | 2455 (42.46) | | 48.54 | |
| Female | 3327 (57.54) | | 51.46 | |
| **Age group (years)** | | | | |
| 18-49 | 2390 (41.34) | | 53.93 | |
| 50-70 | 2246 (38.84) | | 29.72 | |
| 70+ | 1146 (19.82) | | 16.35 | |
| **Residential area** |  | |  | |
| Urban | 3284 (56.80) | | 62.79 | |
| Semi-urban | 1034 (7.88) | | 16.53 | |
| Rural | 1464 (25.32) | | 20.67 | |

Percentages are presented as unweighted N (%) and weighted percentages based on survey weights applied to achieve national representativeness. Minor discrepancies are due to rounding.

**Table S2** Descriptive statistics of the *FS* for Study 2

|  | Mean | Median | SD | Q1 | Q3 |
| --- | --- | --- | --- | --- | --- |
| Phase I | 48.7 | 49 | 4.8 | 45.3 | 52.8 |
| Phase II | 48.5 | 49 | 5.3 | 46 | 53 |

FS Flourishing Scale, SD standard deviation

| **Table S3** Sample frequencies and percentages of each *FS* item at Phase I of Study 2 | | | | | | |  |
| --- | --- | --- | --- | --- | --- | --- | --- |
|  | | | | | | |  |
| ITEM | Strongly disagree | Disagree | Somewhat disagree | Neither agree nor disagree | Somewhat agree | Agree | Strongly agree |
| 1 | 0 (0.0) | 2 (1.0) | 1 (0.5) | 6 (3.0) | 21 (10.5) | 67 (33.5) | 103 (51.5) |
| 2 | 2 (1.0) | 1 (0.5) | 0 (0.0) | 9 (4.5) | 41 (20.5) | 86 (43.0) | 61 (30.5) |
| 3 | 0 (0.0) | 0 (0.0) | 2 (1.0) | 4 (2.0) | 24 (12.0) | 87 (43.5) | 83 (41.5) |
| 4 | 0 (0.0) | 0 (0.0) | 2 (1.0) | 8 (4.0) | 39 (19.5) | 81 (40.5) | 70 (35.0) |
| 5 | 0 (0.0) | 1 (0.5) | 1 (0.5) | 2 (1.0) | 23 (11.5) | 85 (42.5) | 88 (44.0) |
| 6 | 0 (0.0) | 0 (0.0) | 4 (2.0) | 9 (4.5) | 30 (15.0) | 80 (40.0) | 77 (38.5) |
| 7 | 0 (0.0) | 1 (0.5) | 6 (3.0) | 15 (7.5) | 35 (17.5) | 79 (39.5) | 64 (32.0) |
| 8 | 0 (0.0) | 1 (0.5) | 3 (1.5) | 12 (6.0) | 33 (16.5) | 95 (47.5) | 56 (28.0) |

Values are presented as n (%). FS Flourishing Scale.

| **Table S4** Sample frequencies and percentages of each *FS* item at Phase II of Study 2 | | | | | | |  |
| --- | --- | --- | --- | --- | --- | --- | --- |
|  | | | | | | |  |
| ITEM | Strongly disagree | Disagree | Somewhat disagree | Neither agree nor disagree | Somewhat agree | Agree | Strongly agree |
| 1 | 0 (0.0) | 2 (1.0) | 1 (0.5) | 5 (2.5) | 23 (11.5) | 60 (30.0) | 109 (54.5) |
| 2 | 0 (0.0) | 4 (2.0) | 3 (1.5) | 10 (5.0) | 39 (19.5) | 94 (47.0) | 50 (25.0) |
| 3 | 0 (0.0) | 0 (0.0) | 1 (0.5) | 8 (4.0) | 23 (11.5) | 89 (44.5) | 79 (39.5) |
| 4 | 0 (0.0) | 2 (1.0) | 3 (1.5) | 7 (3.5) | 34 (17.0) | 84 (42.0) | 70 (35.0) |
| 5 | 0 (0.0) | 1 (0.5) | 0 (0.0) | 4 (2.0) | 31 (15.5) | 68 (34.0) | 96 (48.0) |
| 6 | 0 (0.0) | 1 (0.5) | 3 (1.5) | 9 (4.5) | 30 (15.0) | 80 (40.0) | 77 (38.5) |
| 7 | 0 (0.0) | 3 (1.5) | 0 (0.0) | 19 (9.5) | 39 (19.5) | 80 (40.0) | 59 (29.5) |
| 8 | 0 (0.0) | 1 (0.5) | 4 (2.0) | 13 (6.5) | 36 (18.0) | 88 (44.0) | 58 (29.0) |

Values are presented as n (%). FS Flourishing Scale.


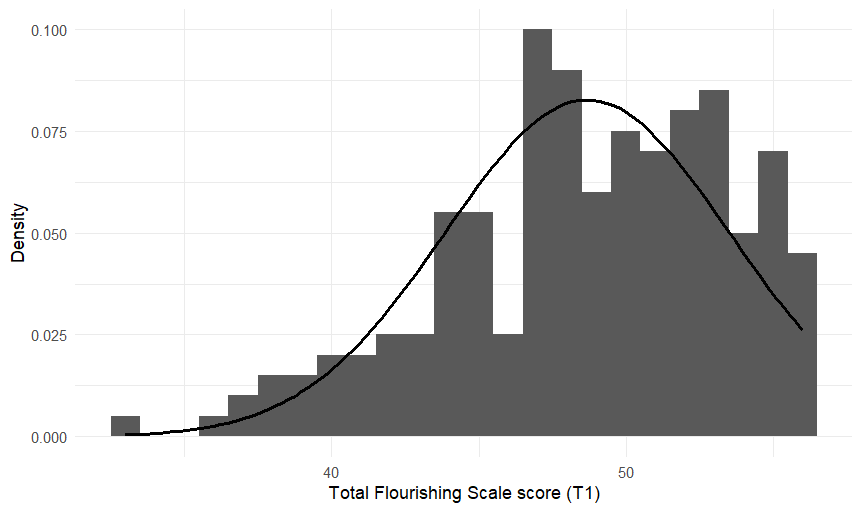


**Fig. S1.** Distribution of total *Flourishing Scale* scores at Phase I of Study 2.
The solid line represents the normal distribution based on the sample mean and standard deviation.

**
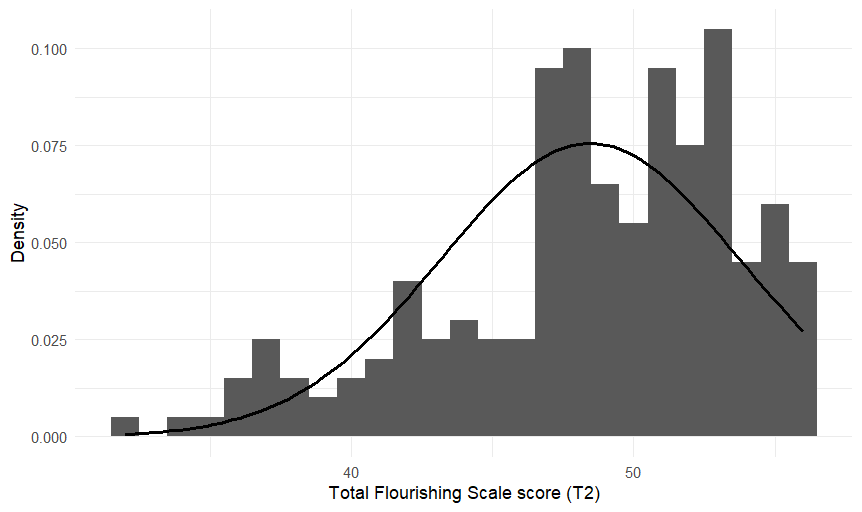
**

**Fig. S2.** Distribution of total *Flourishing Scale* scores at Phase II of Study 2.
The solid line represents the normal distribution based on the sample mean and standard deviation.

**
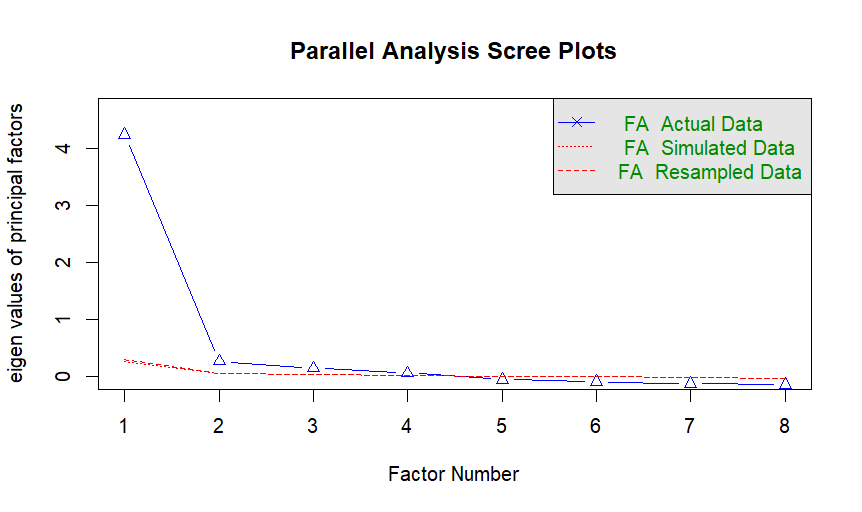
**

**Fig. S3.** Parallel analysis results (Scree plot): observed eigenvalues (blue) compared with simulated random eigenvalues (red) to determine factor retention of Study 1.
